# In Vitro Antioxidant, Anti-Platelet and Anti-Inflammatory Natural Extracts of Amphiphilic Bioactives from Organic Watermelon Juice and Its By-Products

**DOI:** 10.3390/metabo16010081

**Published:** 2026-01-19

**Authors:** Emmanuel Nikolakakis, Anna Ofrydopoulou, Katie Shiels, Sushanta Kumar Saha, Alexandros Tsoupras

**Affiliations:** 1 Hephaestus Laboratory, School of Chemistry, Faculty of Sciences, Democritus University of Thrace, St. Lukas, 65404 Kavala, Greece; manosgnikolakakis@gmail.com (E.N.); anofrid@chem.duth.gr (A.O.); 2 Centre for Applied Bioscience Research, Technological University of the Shannon: Midlands Midwest, Moylish Park, V94 E8YF Limerick, Ireland; katie.shiels@tus.ie (K.S.); sushanta.saha@tus.ie (S.K.S.)

**Keywords:** *Citrullus lanatus*, watermelon, by-products, antioxidant, anti-inflammatory, anti-platelet, polar lipids, amphiphilic bioactives, phenolic, carotenoid

## Abstract

Background/Objectives: Watermelon (*Citrullus lanatus*) processing generates substantial quantities of rind, seeds, and residual pulp that are typically discarded despite being rich in polyunsaturated fatty acids, polar lipids, carotenoids, and phenolic compounds. These amphiphilic bioactives are increasingly recognized for their roles in modulating oxidative stress, inflammation, and platelet activation; however, the lipid fraction of watermelon by-products remains insufficiently characterized. This study examined organic watermelon juice and its by-products to isolate, characterize, and evaluate extracts enriched in amphiphilic and lipophilic bioactives, with emphasis on their in vitro antioxidant, anti-inflammatory, and antithrombotic properties. Methods: total lipids were extracted using a modified Bligh–Dyer method and fractionated into total amphiphilic compounds (TAC) and total lipophilic compounds (TLC) via counter-current distribution. Phenolic and carotenoid levels were quantified, and antioxidant capacity was assessed using DPPH, ABTS, and FRAP assays. Anti-platelet and anti-inflammatory activities were evaluated against ADP- and PAF-induced platelet aggregation. Structural characterization of polar lipids was performed using ATR–FTIR, and LC–MS was used to determine fatty acid composition and phospholipid structures. Results and Discussion: Carotenoids were primarily concentrated in the TLC fractions with high ABTS values for antioxidant activity, while phenolics mostly in the juice, the TACs of which showed the strongest total antioxidant capacity based on DPPH. TAC fractions of both samples showed also higher FRAP values of antioxidant activity, likely due to greater phenolic content. TAC extracts also exhibited notable inhibition of PAF- and ADP-induced platelet aggregation, associated with their enriched ω-3 PUFA profiles and favorable ω-6/ω-3 ratios based on their LC-MS profiles. Conclusions: Overall, watermelon products (juice) and by-products represent a valuable and sustainable source of amphiphilic bioactives with significant antioxidant, anti-inflammatory, and anti-platelet potential, supporting their future use in functional foods, nutraceuticals, and cosmetic applications.

## 1. Introduction

Watermelon (*Citrullus lanatus*) is one of the most widely cultivated fruits worldwide, generating substantial quantities of seeds and processing residues that are commonly discarded. This represents a considerable loss within modern sustainability and circular-economy frameworks, as watermelon seeds contain 30–40% oil enriched in polyunsaturated fatty acids, polar lipids, carotenoids, tocopherols, and phenolic compounds [1]. Their recovery and characterization have therefore become increasingly relevant, particularly in the context of the expanding evidence linking oxidative stress, chronic inflammation, and platelet activation to the development of cardiometabolic and thrombo-inflammatory diseases [2].

Amphiphilic lipid molecules such as phospholipids and glycolipids have attracted attention for their ability to modulate biological pathways related to oxidative balance and inflammation [3]. Several studies have demonstrated that dietary polar lipids from several natural sources, including fruits and their by-products, can inhibit platelet-activating factor (PAF) receptor signaling, reduce platelet aggregation, and suppress inflammatory mediator production [4,5,6]. Similarly, carotenoid- and PUFA-rich lipid matrices exhibit antioxidant and membrane-protective activities, underscoring their relevance as functional ingredients [4,5,6].

Despite this emerging body of research, the lipid fraction of watermelon by-products remains underexplored. Existing studies primarily focus on crude seed oil, with limited insight into how solvent polarity shapes extractable lipid classes, how amphiphilic fractions differ from neutral lipid extracts, or how these fractions influence biological activities such as antioxidant capacity and inhibition of platelet activation [7].

The lack of comprehensive structural and functional characterization represents a major gap, particularly given the potential of watermelon processing waste as a low-cost, sustainable source of bioactive lipids. Understanding solvent-specific extraction behavior is essential for optimizing recovery of bioactive compounds and for establishing structure–activity relationships relevant to functional food or nutraceutical applications.

In this context, the present study aims to isolate, characterize, and evaluate the in vitro biological activities of total lipophilic (TLC) and total amphiphilic (TAC) lipid extracts obtained from organic watermelon flesh and by-products. Specifically, we assess their phenolic and carotenoid content, antioxidant capacity (via DPPH, ABTS, and FRAP assays), and biological activity against thrombo-inflammatory mediators such as PAF and ADP. Structural elucidation using ATR-FTIR spectroscopy and LC-MS analysis is further employed to establish structure–activity relationships between the identified lipid species and their biofunctional properties. Overall, this work provides new insights into the potential utilization of watermelon by-products as sustainable sources of bioactive metabolites for functional food and nutraceutical applications.

## 2. Materials and Methods

### 2.1. Materials, Reagents and Instrumentation

All solvents used for extraction and analysis, including methanol (99.8%), ethanol (96.6%), n-octane, petroleum ether, and chloroform (99.8%), were of analytical grade and were purchased from Sigma Aldrich (St. Louis, MO, USA). Reagents employed in the assays comprised Tris(hydroxymethyl)aminomethane (Tris), hydrochloric acid (HCl), sodium carbonate (Na_2_CO_3_), 2,2-diphenyl-1-picrylhydrazyl (DPPH), 2,2′-azino-bis(3-ethylbenzothiazoline-6-sulfonic acid) (ABTS), sodium persulfate, acetic acid, and sodium acetate. Standards for phenolic compounds (gallic acid, catechin, and quercetin), polar lipids (soy phospholipids), carotenoids (β-carotene), and the antioxidant reference compound 6-hydroxy-2,5,7,8-tetramethylchroman-2-carboxylic acid (Trolox) were purchased from Sigma-Aldrich (St. Louis, MO, USA). The rotary BUCHI flash rotary evaporator was purchased from Alfa Analytical Instruments Ltd. (Representative of BUCHI in Greece, Gerakas, Attiki, Greece).

All spectrophotometric measurements were performed using a uniSPEC 2 UV–Vis spectrophotometer (LLG Labware, Meckenheim, Germany). Structural characterization of samples and reference standards was carried out using attenuated total reflectance Fourier-transform infrared spectroscopy (ATR-FTIR) on a PerkinElmer Frontier ATR/FT-NIR/MIR spectrometer (PerkinElmer, Waltham, MA, USA).

Blood samples were collected with 20-gauge safety needles in evacuated sodium citrate S-monovettes^®^ (Sarstedt Ltd., Wexford, Ireland). Experiments were conducted exclusively with platelet-rich plasma (PRP) prepared from healthy volunteers. The study was approved by the Ethics Committee of Democritus University of Thrace (protocol code ΔΠΘ/ΕHΔΕ/7690/70, 27 September 2024), and informed consent was obtained from all donors.

Human PRP (hPRP) was prepared for platelet function assays using a Chrono-log 490 four-channel strobilometric platelet aggregometer (Chrono-log Corp., Havertown, PA, USA) with AGGRO/LINK^®^8 software (Version 8, Chrono-log Corp., Havertown, PA, USA). All consumables were supplied by Chrono-log. Centrifugation steps were performed with a Nahita Blue Medibas + low-speed centrifuge (Auxilab, Beriain, Navarra, Spain) at a maximum speed of 4000 rpm.

### 2.2. Sample Preparation and Extraction-Separations

Three organically cultivated *Citrullus lanatus* (watermelon) fruits were obtained from an organic producer in Crete, Greece, five days prior to extraction and stored in a cool, shaded environment until use. The extraction was performed at room temperature, with an extraction time of 1 h. Lipid extraction was performed using a modified Bligh–Dyer method (1959), as described by Tsoupras et al. (2024) [8], which is suitable for extracting the total lipid content containing the major classes of amphiphilic compounds from natural sources. This procedure enabled the separation of total lipids from non-lipid components.

The lipid fraction, comprising both amphiphilic molecules (e.g., polar lipids and amphiphilic phenolics and carotenoids) and neutral lipids (e.g., triacylglycerols, cholesterol esters, etc.), was further fractionated using a modified counter-current distribution (CCD) technique based on the method of Galanos and Kapoulas, adapted by Tsoupras et al. (2024) [8]. The combination of these two methods provides an efficient and reproducible approach for isolating and separating the more polar (amphiphilic) bioactive compounds from the more neutral lipids from a total lipid substrate, without altering their native composition [4,5,6,8].

Unlike conventional high-temperature extraction techniques such as Soxhlet extraction, this low-temperature approach minimizes the risk of thermal degradation of polyunsaturated fatty acids and other labile bioactives, thereby preserving the integrity and biological activity of the lipid extracts [9].

#### 2.2.1. Preparation of Watermelon Juice Samples

The edible flesh of organic *Citrullus lanatus* fruits was manually separated from the rind and seeds using a stainless-steel knife and processed with a juicer to obtain approximately 40 mL of juice per fruit. Lipid extraction was performed using 100 mL of methanol and 50 mL of chloroform. After brief settling, 50 mL of chloroform and 50 mL of deionized water were added to a separatory funnel, and the lower organic phase was collected. The extraction was repeated three times for each sample to ensure complete recovery of the lipid fraction.

The combined organic phases were transferred to round-bottom flasks, sealed with parafilm and aluminum foil, and evaporated under reduced pressure (270 mbar) using a rotary evaporator to remove chloroform. Residual aqueous (methanol–water) content was removed by adding several drops of ethanol to form an azeotropic mixture, which was evaporated at 70 mbar. The resulting solid residues were redissolved in a 1:1 (*v*/*v*) chloroform–methanol solution and transferred to pre-weighed glass vials. The procedure yielded three independent extracts, designated as J1, J2, and J3.

All samples were gently evaporated under a nitrogen atmosphere to prevent oxidative degradation and subsequently weighed to determine extraction yield.

#### 2.2.2. Preparation of Watermelon By-Product Samples

Approximately 100 g of watermelon rind, with the red mesocarp layer removed, was processed using a juicer. The resulting juice, together with the solid residues (peel and pulp), was transferred to a blender. Methanol and chloroform were added to achieve a juice/methanol/chloroform ratio of 0.8:2:1 (*v*/*v*/*v*). The mixture was filtered under reduced pressure, and the filtrate was extracted with 50 mL of chloroform and 50 mL of deionized water. After 3 h of phase separation, the organic layer was collected and stored in sealed round-bottom flasks.

The extraction procedure was performed three times to obtain replicate samples from different fruits. The organic extracts were evaporated under reduced pressure using a rotary evaporator, and the resulting solid residues were redissolved in chloroform–methanol (1:1, *v*/*v*). The residues were then gently dried under nitrogen to remove any remaining solvent. The final extracts were designated as BP1, BP2, and BP3. Each sample was weighed to calculate the extraction yield.

The extraction yield was calculated as the percentage of dry extract mass obtained relative to the initial mass of the sample. The following equation was used:

Extraction Yield (%) = (mass of dry extract (g)/mass of initial sample (g)) × 100

where

mass of dry extract = the weight of the lipid residue after solvent evaporation

mass of initial sample = the weight of the watermelon juice or by-product before extraction

#### 2.2.3. Counter-Current Distribution (CCD)

Counter-current distribution (CCD) was employed to fractionate the total lipid extracts into the more polar (amphiphilic) and the more neutral (lipophilic) lipid fractions, following a modified Galanos and Kapoulas method as described by Tsoupras et al. (2024) [8]. Pre-equilibrated petroleum ether and 87% ethanol in water were used as the solvent system. Prior to separation, the solvents were equilibrated by partitioning the hydroalcoholic phase into petroleum ether to optimize the partition coefficient and improve separation efficiency.

Two separatory funnels were arranged vertically, each containing 30 mL of equilibrated petroleum ether. To the upper funnel, 10 mL of 87% equilibrated ethanol and the lipid sample were added and mixed thoroughly using a vortex mixer. After phase separation, the lower (more polar) phase from the first funnel was transferred to the second funnel and mixed again. The bottom layer (more polar) phase from the second funnel was collected into a round-bottom flask for further processing.

The remaining sample residue was rinsed with 1 mL of a 1:1 (*v*/*v*) chloroform–methanol solution, filtered through borax, and reintroduced into the first funnel. Subsequently, 10 mL of 87% equilibrated ethanol was added, and the process was repeated several times. The final more neutral organic phases of petroleum ether from both funnels were collected separately and stored for further analysis.

#### 2.2.4. Evaporation of Samples

The combined petroleum ether fractions obtained from the CCD process of a TL sample were evaporated using a rotary evaporator (Büchi Rotavapor, Alfa Analytical Instruments Ltd., Gerakas, Attiki, Greece) under reduced pressure (350 mbar). To remove residual water, 2 mL of ethanol was added, and the azeotropic mixture was evaporated at a pressure ranging from 70 to 50 mbar. The resulting residues were dissolved in petroleum ether and transferred into pre-weighed glass vials. The rotary flasks were rinsed with small volumes of petroleum ether to ensure complete recovery. These petroleum ether-phase samples containing the more neutral and thus lipophilic TACs were designated as N1–N6.

Similarly, the combined hydroalcoholic (polar) fractions were evaporated at 75 mbar. To remove residual water, 2 mL of ethanol was added, and the azeotropic mixture was evaporated at a pressure ranging from 70 to 50 mbar. The solid residues of the more polar amphiphilic TACs were dissolved in a 1:1 (*v*/*v*) chloroform–methanol solution. These samples were designated as P1–P6. All extracts were gently dried under an inert nitrogen atmosphere to prevent oxidation, weighed, and their extraction yields were calculated.

#### 2.2.5. Aliquoting of the Amphiphilic (TAC) and Neutral (TLC) Lipid Samples

For final sample preparation, 1 mL of ethanol was added to each polar extract (P1–P6), and 1 mL of n-octane was added to each neutral extract (N1–N6). All samples were homogenized using a vortex mixer to ensure uniform dispersion.

From each extract, 0.1 mL aliquots were transferred into seven test tubes, leaving 0.3 mL of the original solution in each source vial. This procedure yielded eight subsamples per extract (e.g., N1A–N1H), resulting in a total of 96 samples prepared for subsequent analyses, including chemical composition and bioactivity assays.

### 2.3. Determination of Total Phenolic Content (Folin–Ciocalteu Assay)

The total phenolic content (TPC) of the extracts was determined using the Folin–Ciocalteu (FC) colorimetric method, following the protocol of Tsoupras et al. (2024) [8]. Briefly, 1 mL of deionized water was added to each sample and homogenized using a vortex mixer, followed by the addition of 1 mL of Folin–Ciocalteu reagent. After mixing, the reaction was allowed to proceed for 7 min before adding 3 mL of sodium carbonate (Na_2_CO_3_) solution. The mixture was vortexed again and incubated in the dark for 2 h at room temperature. The absorbance was then measured at 765 nm using a UV–Vis spectrophotometer. Phenolic content was quantified against a gallic acid calibration curve and expressed as milligrams of gallic acid equivalents per gram of extract (mg GAE/g extract).

### 2.4. Determination of Total Carotenoid Content (TCC)

Total carotenoid content (TCC) was determined according to Tsoupras et al. (2024) [8]. Samples were dissolved in 2 mL of n-octane, and absorbance was recorded at 450 nm. Appropriate dilutions were performed as required. Carotenoid concentration was calculated using a β-carotene calibration curve, and results were expressed as milligrams of β-carotene equivalents per gram of extract (mg CE/g extract).

### 2.5. Determination of Total Antioxidant Activity (TAA)

All lipid extracts were assessed for potential antioxidant activities by utilizing three complementary in vitro assays: DPPH radical scavenging, ABTS cation radical decolorization, and ferric-reducing antioxidant power (FRAP), based on the relative methodologies described by Tsoupras et al. (2024) [8].

#### 2.5.1. ABTS Assay

A total of 2 mL of ABTS reagent was added to an aliquot of each sample and the mixture was vortexed and after being left for 7 min of incubation for the reaction to take place, the absorbance of the mixture was then measured at 734 nm. Trolox was used as the positive control antioxidant, and results were expressed as ABTS values, meaning as micromoles of Trolox equivalents per gram of dry weight (μmol TE/g DW) according to the equation:

ABTS (μmol TE/g DW) = c × V × t/m,

with *c* standing for the Trolox concentration (μmol/mL) derived from a standard curve for the concentration dependent antioxidant capacity of Trolox, *V* standing for the sample volume (mL), *t* standing for the dilution factor, and *m* standing for the sample dry weight (g).

#### 2.5.2. DPPH Assay

In each sample 1 mL of Tris–HCl buffer (pH 7.5) was added and the mixture was vortexed, and then 1 mL of DPPH reagent was added, and the mixture was again vortexed thoroughly. After being left for 30 min of incubation for the reaction to take place, the absorbance of the mixture was then measured at 517 nm, and the radical scavenging activity (%) was calculated according to the equation:

Inhibition (%) = (A1 − A2) × 100/A1,

where *A*_1_ stands for the absorbance of the control and *A*_2_ for the absorbance of the sample.

The concentration required to inhibit 50% of the DPPH radical (IC_50_) was determined, and results were expressed as Trolox equivalent antioxidant capacity (TEAC) by comparing the IC50 value of the sample with that of the Trolox, according to the formula:

TEAC = IC50 of Trolox (μg/L)/IC50 of the sample (μg/L).

#### 2.5.3. FRAP Assay

A total of 3 mL of FRAP working solution was added to each sample and vortexed. After 15 min of incubation for the reaction to take place, the absorbance of the mixture was measured at 593 nm, with Trolox being the positive control standard. Results were expressed as FRAP values, meaning as μmol TE/g DW according to the equation:

FRAP (μmol TE/g DW) = c × V × t/m,

with *c* standing for the Trolox concentration (μmol/mL) derived from a standard curve for the concentration dependent antioxidant capacity of Trolox, *V* standing for the sample volume (mL), *t* standing for the dilution factor, and *m* standing for the sample dry weight (g).

### 2.6. Platelet Aggregometry Biological Assays

The antiplatelet and anti-inflammatory activities of watermelon juice (WMJ) and watermelon by-product (WMBP) lipid extracts were evaluated according to the protocol of Kosidou et al. (2025) [10]. The method is based on monitoring permeability (optical density) changes in platelet-rich plasma (PRP) following activation by specific agonists, in the presence or absence of bioactive extracts.

Platelet aggregation was induced using either adenosine diphosphate (ADP) or platelet-activating factor (PAF) as agonists. ADP activates platelets via receptor-mediated pathways associated with thrombosis [11], while PAF serves as a key mediator of inflammation, allowing simultaneous assessment of the extracts’ antithrombotic and anti-inflammatory potential [12].

Blood samples were collected individually from *n* = 6 different healthy adult volunteers after an 8 h fast. PRP was prepared by centrifugation at 194× *g* for 18 min at 24 °C, and the supernatant was carefully collected. Platelet-poor plasma (PPP) was obtained by centrifuging the remaining sample at 1465× *g* for 20 min at 24 °C.

Aliquots of 250 µL PRP were transferred to glass cuvettes of a four-channel optical aggregometer (Born type, Havertown, PA, USA) equipped with AGGRO/LINK software (Version 8, Chrono-log Corp., Havertown, PA, USA) and magnetic stirring at 1200 rpm. PPP served as the reference for 100% light transmission. Calibration was performed by setting PRP to 0% and PPP to 100% transmission, following the manufacturer’s instructions. A stable baseline was recorded for 30 s prior to agonist addition.

Platelet aggregation was induced by adding a defined concentration of PAF or ADP, and the resulting increase in light transmission was recorded to generate aggregation curves. The maximal reversible aggregation induced by the agonist alone was defined as 100% aggregation (0% inhibition). To assess inhibitory activity, PRP was incubated with varying concentrations of lipid extract before agonist addition. A decrease in aggregation response indicated inhibition of platelet activation.

The half-maximal inhibitory concentration (IC_50_) was calculated for each extract, corresponding to the amount (µg) required to achieve 50% inhibition of PAF- or ADP-induced platelet aggregation in 250 µL PRP. Lower IC_50_ values indicated stronger inhibitory potency and greater antithrombotic potential.

### 2.7. Assessment of Fatty Acid Composition by LC–MS

TAC amphiphilic lipid extracts from watermelon juice and from watermelon by-products were further analyzed in triplicate (*n* = 3) using liquid chromatography–mass spectrometry (LC–MS) to determine their structural composition and free fatty acid (FFA) profiles, as described by Tsoupras et al. (2024) [8].

Each sample was divided into two equal portions and dried under nitrogen. One portion was subjected to saponification using 1.5 mL of 2.5 M KOH in methanol (1:4, *v*/*v*), vortexed, and incubated at 72 °C for 15 min. After cooling, 225 µL of formic acid, 1.725 mL of chloroform, and 375 µL of ultrapure water were added. The mixture was vortexed and phase-separated; the lower chloroform layer containing FFAs was collected, evaporated to dryness, and stored at −20 °C.

For LC–MS analysis, dried lipid residues were reconstituted in 500 µL methanol/dichloromethane (2:1, *v*/*v*), centrifuged at 13,000 rpm for 6 min (Heraeus Biofuge Stratos, Fisher Scientific, Dublin, Ireland), and filtered through 3 kDa ultrafiltration units (Amicon Ultra, Merck Millipore, Ireland). A 10 µL aliquot was injected into an Agilent 1260 HPLC system coupled to a Q-TOF mass spectrometer (Agilent 6520, Agilent, Agilent Technologies Ireland Ltd., Cork, Ireland)) equipped with an electrospray ionization (ESI) source. Separation was achieved on an Agilent Poroshell 120 C18 column (2.7 µm, 3.0 × 150 mm) (Agilent, Agilent Technologies Ireland Ltd., Cork, Ireland).

The mobile phases consisted of 2 mM ammonium acetate in water (A) and 2 mM ammonium acetate in 95% acetonitrile (B). The gradient program began at 60% B for 1 min, increased to 90% B in 2.5 min, held for 1.5 min, then ramped to 100% B over 0.5 min and maintained for 4 min before re-equilibration. Flow rate was initially 0.3 mL/min, increased to 0.6 mL/min at 10 min, and maintained thereafter.

The MS was operated in negative ion mode (scan range 0–1100 *m*/*z*), with a drying gas flow of 5 L/min, nebulizer pressure of 30 psi, and temperature of 325 °C. The fragmentor and skimmer voltages were set to 175 V and 65 V, respectively, and capillary voltage to 3500 V. Reference ions at *m*/*z* 1033.988 and *m*/*z* 112.9855 were used for mass calibration.

Identification of FFA and phospholipid species was achieved through survey, daughter, precursor, and neutral loss scans. Lipid structures were confirmed using the LIPID MAPS: Nature Lipidomics Gateway database (www.lipidmaps.org, accessed on 10 January 2026), considering both experimental delta values and LC–MS results of saponified FFAs, as previously described by Tsoupras et al. (2024) [8].

### 2.8. ATR–FTIR Analysis

Attenuated Total Reflectance Fourier-Transform Infrared (ATR–FTIR) spectroscopy was employed to characterize the chemical composition of lipid extracts. In this technique, an infrared beam passes through a high-refractive-index crystal, interacting with the sample in contact with its surface. Absorbed wavelengths induce molecular vibrations, producing characteristic spectra that reveal functional groups and molecular structures.

For analysis, each extract was dissolved in isopropanol and applied directly to the ATR crystal plate of a PerkinElmer Frontier ATR/FT-NIR/MIR spectrometer, following the procedure described by Vordos et al. (2018) [13]. The pressure tip was adjusted to ensure full contact, and spectra were collected over a wavenumber range of 4000–600 cm^−1^.

Standard compounds (quercetin, catechin, gallic acid, β-carotene, and soy-derived polar lipids) were analyzed under identical conditions for spectral comparison. Isopropanol spectra were also recorded to correct for solvent interference. The obtained spectra were examined for characteristic absorption peaks corresponding to phenolic, carotenoid, and lipid functional groups.

### 2.9. Statistical Analysis

All bioactivity experiments—including antioxidant assays and platelet aggregation inhibition assays—were performed using three independent biological replicates (*n* = 3). For the anti-inflammatory and antiplatelet assessments, each of the three extracts was tested in blood samples obtained from several individual healthy donors, yielding a total of nine measurements per extract (N = 9).

Data normality was evaluated using the Kolmogorov–Smirnov test. Variables that met the assumption of normality (IC_50_ values for anti-inflammatory and antiplatelet activity, as well as fatty acid composition) were analyzed using one-way ANOVA, followed by LSD post hoc comparisons. These results are reported as mean ± standard deviation (SD). For datasets that were non-normally distributed (phenolic content, carotenoid content, and antioxidant activity), the Kruskal–Wallis test was applied, and results are presented as minimum, maximum, and median values. Statistical comparisons were performed both between sample types (e.g., juice vs. by-products) and between extraction fractions (TAC vs. TLC). A significance threshold of *p* < 0.05 was used in all analyses.

## 3. Results

### 3.1. Yield of Extraction

The extraction yields for each sample type are summarized in Table 1.

### 3.2. Total Phenolic Content

The total phenolic content (TPC) for each TAC and TLC fraction of each sample type (juice and by-products) are summarized in Table 2.

### 3.3. Total Carotenoid Content

The total carotenoid content (TCC) for each TAC and TLC fraction of each sample type (juice and by-products) are summarized in Table 3.

### 3.4. Antioxidant Activity Expressed as ABTS Values

The obtained antioxidant activity based on the ABTS assay for each TAC and TLC fraction of each sample type (juice and by-products) are expressed as ABTS values and are summarized in Table 4.

### 3.5. Antioxidant Activity Expressed as TEAC Values According to the DPPH Assay

The obtained total antioxidant capacity based on the DPPH assay for each TAC and TLC fraction of each sample type (juice and by-products) are summarized in Table 5.

### 3.6. Antioxidant Activity Expressed as FRAP Values

The obtained antioxidant activity based on the FRAP assay for each TAC and TLC fraction of each sample type (juice and by-products) are expressed as FRAP values and are summarized in Table 6.

### 3.7. ATR-FTIR Spectroscopy

Characteristic infrared (IR) absorption peaks for the TAC extracts from both the watermelon juice and its by-products, with corresponding functional group assignments are summarized in Table 7.

### 3.8. Platelet Aggregometry Assay—Evaluation of Antithrombotic and Anti-Inflammatory Activity

**Figure 1:**
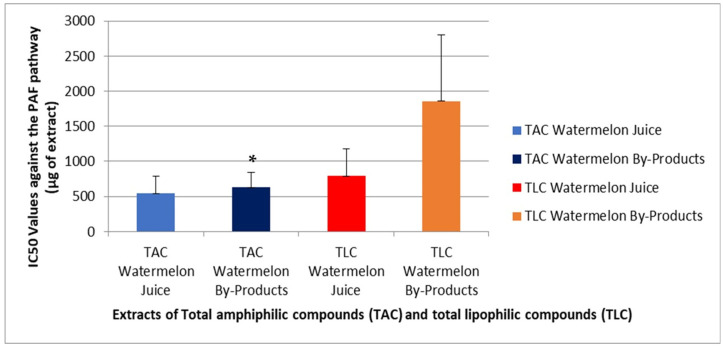
Anti-inflammatory effects of the TAC and TLC fractions from watermelon juice and its by-products against the PAF pathway. Results are expressed as the IC50 value for each sample (compound/extract/fraction), which equals to the mass in μg of the sample that when present in the aggregometer cuvette with the 250 μL of hPRP, it can cause 50% of inhibition of the PAF-induced inflammatory activation and aggregation of hPRP (The lower the IC50 value the more potent the anti-inflammatory activity for an extract). * denotes statistically significant differences in the more potent anti-inflammatory activity against PAF (lower IC50 values) of the TAC fraction from the by-products compared to the TLC fractions of this sample (*p* < 0.05 in this comparison according to the ANOVA test assessment in *n* = 6 blood samples from different donors). Abbreviations: TAC = the fraction with the total amphiphilic compounds; TLC = the fraction with the total lipophilic compounds; hPRP = human plasma rich platelets; IC50 value = half maximum inhibitory concentration.

**Figure 2:**
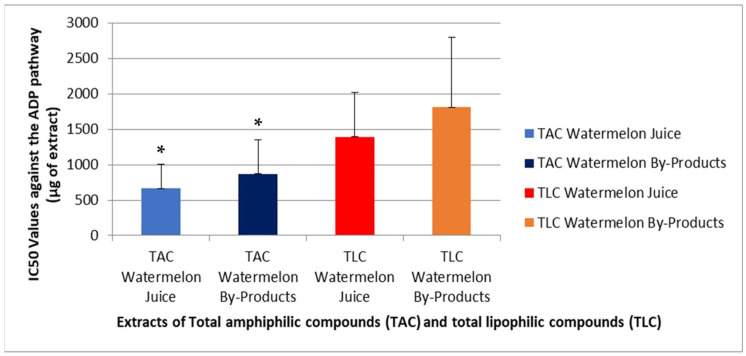
Anti-platelet effects of the TAC and TLC fractions from watermelon juice and its by-products against the ADP pathway. Results are expressed as the IC50 value for each sample (compound/extract/fraction), which equals the mass in μg of the sample that when present in the aggregometer cuvette with 250 μL of hPRP can cause 50% of inhibition of the ADP-induced aggregation of hPRP (again, the lower the IC50 value the more potent the anti-platelet activity for the sample). * denotes statistically significant difference in the more potent anti-platelet activity against ADP (lower IC50 values) of the TAC fraction from the juice and the by-products compared to the TLC fractions for each of these samples assessed (*p* < 0.05 in all these comparisons according to the ANOVA test and Tukey post hoc assessment in *n* = 6 blood samples from different donors). Abbreviations: TAC = the fraction with the total amphiphilic compounds; TLC = the fraction with the total lipophilic compounds; hPRP = human plasma rich platelets; IC50 value = half maximum inhibitory concentration.

Figure 1 and Figure 2 present the results of the platelet aggregation assays using platelet-activating factor (PAF) and adenosine-5′-diphosphate (ADP) as agonists for inducing the aggregation of hPRP, respectively.

### 3.9. Fatty Acid Composition of the Saponified TAC Fractions from Watermelon Juice and By-Products

The fatty acid compositions of the bioactive polar lipids obtained by the LC–MS analysis of the saponified TAC fractions from both the Watermelon juice and its by-products are shown in Table 8.

### 3.10. LC-MS Analysis of the Non-Saponified TAC Extracts from Both the Watermelon Juice and Its By-Products

**Figure 3:**
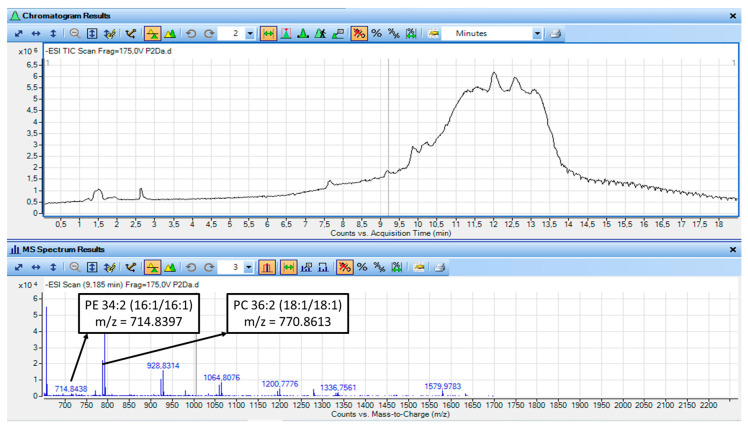
A representative chromatogram and LC-MS analysis of the non-saponified TACs from Watermelon Juice, with representative identified polar lipids in the specific elution time.

**Figure 4:**
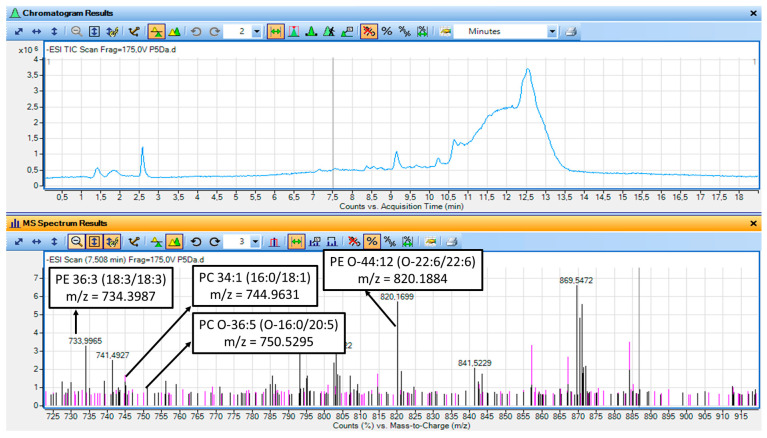
A representative chromatogram and LC-MS analysis of the non-saponified TACs from Watermelon By-Products, with representative identified polar lipids in the specific elution time.

Representative LC_MS Chromatograms and MS spectra of the LC-MS analysis of the non-saponified TAC fractions from watermelon juice and its by-products are shown in Figure 3 and Figure 4.

In addition, the representative molecular species of the polar lipids of the non-saponified TAC fractions from watermelon juice and its by-products, along with elution time of this analysis, their *m*/*z*/ and delta values obtained from https://www.lipidmaps.org/, accessed on 10 January 2026 and the final proposed structures for each species are shown in Table 9.

## 4. Discussion

### 4.1. Yield of Extraction in TACs and TLCs for Both the Watermelon Juice and the By-Products

The total lipid extraction was performed using a modified Bligh–Dyer method [8], which is well-suited for obtaining intact the full scale of all lipid classes in a total lipid extract from natural sources, including samples with relatively low lipid content such as watermelon (*Citrullus lanatus*) tissues and by-products. All total amphiphilic compound (TAC) and total lipophilic compound (TLC) extracts were initially screened to identify those exhibiting significant biological activity, after which targeted analyses were conducted on selected fractions.

Consistent with previous studies [1,2], the TL of the watermelon by-products (seed, rind, remnants of flesh, etc.) seems to contain higher content of lipophilic molecules than amphiphilic ones, primarily due to the oily composition of the seeds. Nevertheless, since rind and the flesh remnants are also rich in structural polysaccharides such as cellulose and pectin, in which several amphiphilic compounds are easily trapped during juicing, it seems that the by-products also contain a considerable amount of amphiphilic compounds, which we managed obtain from these polysaccharide “traps” by the specific extraction and separation processes applied. Thus, no statistical differences were observed between the TAC and the TLC contents of the by-products.

In contrast, the juice that was obtained by juicing the flesh contained higher proportions of amphiphilic compounds, including polar lipids like phospholipids and glycolipids, since the juice is a watery substance, in which the lipophilic compounds are not easily dissolved, and if any is detected this may be due to it being trapped at the center of any spontaneously forming micelles formed by the polar lipids or being bound to lipoproteins transferred in such a watery polar environment.

Overall, and as shown in Table 1, extraction of amphiphilic lipids from watermelon juice (WMJ) samples was generally more efficient than that of neutral lipids. For by-product (WMBP) samples, however, the proportions of amphiphilic and neutral lipid fractions were approximately comparable. This can be attributed to the heterogeneous composition of the by-products, which include both seed material—rich in neutral, oil-based lipids—and rind, which contributes amphiphilic constituents. These results are in agreement with trends reported in the literature for plant matrices of mixed composition.

A comparison of total lipid extraction yield between juice and by-product samples (Table 1) revealed no statistically significant difference (*p* > 0.05). Nevertheless, a slight trend toward higher extraction efficiency in juice samples was observed. This may reflect the presence of seed-derived lipophilic molecules in the juice matrix, exceeding the levels typically expected for low-fat samples extracted using the specific extraction and separation process [8].

### 4.2. Total Phenolic Content of Both TACs and TLCs from Watermelon Juice and Its By-Products

Phenolic compounds are a broad class of aromatic organic molecules characterized by the presence of one or more hydroxyl (–OH) groups bonded to an aromatic ring. Numerous phenolic compounds occur naturally, most of which are structurally complex and diverse secondary metabolites synthesized by plants as defense mechanisms against pests, microbial infections, oxidative stress, and ultraviolet radiation [14].

Phenolics have traditionally been described as polar compounds; however, this classification has been re-examined. Many phenolic compounds display limited solubility in water and greater solubility in alcoholic solutions or more lipophilic solvent systems, such as mixtures of alcohols with less polar organic solvents like chloroform. While simple phenols are generally more hydrophilic, more complex phenolic structures—such as flavonoids—exhibit pronounced amphiphilic behavior due to the coexistence of hydrophilic and lipophilic regions. This dual character enables them to interact with both aqueous environments and lipid structures, including cell membranes [14].

The hydroxyl groups of phenolic compounds contribute to polarity through the geometry and polarization of the C–OH bond, allowing hydrogen bond formation with electronegative atoms such as oxygen or nitrogen in polar media, thereby enhancing solubility in polar solvents. In contrast, the aromatic ring confers lipophilic properties that facilitate interactions with nonpolar lipid molecules. As a result, the overall amphiphilicity of phenolic compounds allows their simultaneous association with both hydrophilic and lipophilic phases. This characteristic underlies their functional roles in emulsification, drug delivery systems, and membrane interactions, particularly in food and cosmetic applications.

Due to their redox activity and antioxidant function in plants, phenolic compounds were initially thought to exert health benefits in humans primarily through antioxidant mechanisms. However, their limited bioavailability in aqueous environments and their extensive interactions with various biological molecules—including proteins, enzymes, transcription factors, vitamins, lipoproteins, and lipids—particularly in amphiphilic systems, indicate that their mechanisms of action in vivo are far more complex [14,15]. Consequently, assessing the total phenolic content of a sample provides valuable insight into its potential nutritional value and possible health-promoting effects.

Given this dual nature, phenolic compounds in watermelon and its by-products are expected to be predominantly present in the amphiphilic lipid extracts, whereas neutral lipid extracts would typically contain lower concentrations. The amphiphilic extracts of watermelon juice were therefore anticipated to exhibit the highest phenolic content, owing to the presence of both flavonoids and phenolic acids [16]. Similarly, the amphiphilic extracts of watermelon by-products were expected to contain moderate levels of phenolics [17,18,19,20]. In contrast, the neutral extracts derived from both juice and by-products were anticipated to have minimal phenolic content.

Interestingly, the results of the Folin–Ciocalteu assay (Table 2) supported the fact that both sources are rich in phenolics, while the statistical analysis indicated no significant difference between the TAC and TLC fractions in either juice or by-products. This outcome suggests that the lipid matrix of watermelon may entrap phenolic compounds within the neutral (TLC) fraction, thereby masking the expected polarity-based distribution. Such lipid–phenolic interactions could contribute to the observed bioactivity of both extract types, underscoring the complex interplay between phenolic compounds and the lipid environment.

### 4.3. Total Carotenoid Content in the Extracts from Both the Watermelon Juice and Its By-Products

Carotenoids constitute a large and diverse class of naturally occurring tetraterpene pigments, synthesized by plants, algae, certain bacteria, fungi, and invertebrates, but not by mammals; consequently, their availability in mammals depends entirely on dietary intake [21,22]. Fruits and vegetables account for approximately 80–90% of total carotenoid consumption, while animal-derived products such as milk, cheese, egg yolk, and butter contribute the remaining 10–20% [22]. Several carotenoids have been detected in human plasma like the α-carotene, β-carotene, β-cryptoxanthin, lycopene, lutein, and zeaxanthin, which have been strongly associated with health benefits [22]. Consequently, these compounds have been the primary focus of scientific research within this family.

Furthermore, the extended conjugated double-bond system characteristic of carotenoids confers potent antioxidant and free radical–scavenging properties [22]. These activities underpin their immune-supporting functions and their protective roles against cardiovascular diseases and ultraviolet radiation [22]. Moreover, carotenoids containing one or more β-ionone rings, such as α-carotene, β-carotene, and β-cryptoxanthin, exhibit significant provitamin A activity, making adequate dietary intake essential for maintaining sufficient vitamin A levels [21,22]. Apart from that, several recent studies have associated carotenoids with several other health benefits, including anti-inflammatory and immune system modifiers [21,22], thereby highlighting why the total carotenoids content is important to be fully assessed in a sample due to carotenoids being a nutritionally valuable and biologically important class of compounds with several health promoting properties.

Such bioactive Carotenoids, including lycopene, β-carotene, and phytoene, are abundant in watermelon and especially in the red-fleshed portion of the fruit, and they contribute to its characteristic pigmentation and antioxidant properties [23,24,25]. In contrast, watermelon by-products—including the peel and seeds—contain markedly lower concentrations of these pigments [23,24,25]. Carotenoids are generally distributed both in the neutral lipid (TLC) extracts and in the amphiphilic (TAC) fractions, based on the experimental conditions of the CCD applied. The TLC fractions mostly migrate the more lipophilic carotenoids that lack polar groups and are of high molecular weights, suggesting big hydrocarbon chains, which are usually the most abundant carotenoids, while in the TAC fractions migrate mostly the more amphiphilic carotenoids that contain a polar group with a heteroatom like oxygen and are usually of smaller molecular weights and hydrocarbon chains, which are usually the less abundant carotenoids [26].

The total carotenoid content analysis (Table 3) confirmed this expectation in the juice samples, showing that the TLC extracts of watermelon juice contained significantly higher carotenoid levels than all the other samples (*p* < 0.05 in all these comparisons according to the Kruskal–Wallis test). This result aligns with the high lycopene content typical of watermelon flesh [25]. In the case of by-product samples, carotenoids were found to be more evenly distributed between the TLC and TAC extracts. The presence of measurable carotenoid content in the amphiphilic fractions can be attributed to the interaction of these hydrophobic molecules with amphiphilic lipids, such as phospholipids and glycolipids. These molecules can form stable complexes with both hydrophilic and lipophilic components, allowing partial entrapment and stabilization of carotenoids within the amphiphilic matrix—an effect more pronounced than initially anticipated.

Carotenoids, vitamin A, and its vitaminoids have emerged as pivotal bioactive components in several extracts and/or products infused by such extracts rich in carotenoids, especially in the cosmetic sector due to their potent antioxidant and anti-inflammatory activities, which highly contribute to specific skin-related functions, including their anti-aging, skin regeneration, wound healing, hyperpigmentation, and acne treatment health-promoting effects [22]. Thus, the presence of considerable amounts of carotenoids in the TAC extracts of watermelon by-products further supports their putative utilization as sustainable sources of these bioactive compounds, in a circular economy context.

### 4.4. Antioxidant Activity of Extracts Expressed as ABTS Values

From the outcomes obtained from the ABTS assay that was conducted in both the TAC and the TLC extracts, a considerably high antioxidant activity was found in the amphiphilic (TAC) extracts of both the watermelon juice and its by-products, as the flesh is particularly rich in potent antioxidants such as flavonoids and polyphenolic compounds, as well as some carotenoids, which match with the general concept of antioxidant phytochemicals in fruits and their by-products. Interestingly, the TLC fractions showed higher antioxidant capacity compared to that of the TAC extracts in both juice and by-products (*p* < 0.05 in both these comparisons, according to the Kruskal–Wallis test), probably due to their greater carotenoid concentration—particularly lycopene—which is well documented for its strong radical-scavenging properties in the ABTS assay [27,28] (Table 4).

The effect was especially pronounced in the TLC extracts of the juice, corresponding to the substantially higher carotenoid concentration present in the watermelon flesh relative to its peel and seeds. These findings are consistent with literature reports indicating that carotenoids, particularly lycopene, possess strong free radical–scavenging capacity and play a key role in the antioxidant defense system of plant-derived lipid fractions [28].

### 4.5. Antioxidant Activity of Extracts Expressed as TEAC Values According to the DPPH Assay

From the outcomes obtained based on the DPPH assay that was conducted in both the TAC and the TLC extracts, a considerably high total antioxidant capacity was found in the amphiphilic (TAC) extracts of both the watermelon juice and its by-products, compared to that of a positive standards (Trolox; a synthetic hydrophilic analogue of vitamin E), as the flesh is particularly rich in potent antioxidants such as flavonoids and polyphenolic compounds, which match with the general concept of antioxidant phytochemicals in fruits and their by-products. Previous studies [28,29] have demonstrated that phenolic compounds, owing to their hydroxyl groups and aromatic structures, serve as efficient hydrogen donors capable of neutralizing DPPH radicals. In contrast, carotenoids such as lycopene generally show lower activity in this assay. However, lycopene derived from watermelon has been reported to exhibit stronger radical-scavenging ability than that from other sources, such as tomato [27], suggesting a possible matrix or structural influence on its bioactivity.

At this point it is important to stress out that using TEAC (Trolox Equivalent Antioxidant Capacity) instead of just % inhibition values in the DPPH assay is often better because TEAC provides a more comprehensive, standardized measure of total antioxidant power by comparing your sample’s scavenging ability to a known antioxidant (Trolox), allowing for direct comparison across different samples, while % inhibition only shows a snapshot at a specific concentration, potentially missing the full picture, especially for samples with different reaction kinetics or solubility issues. TEAC accounts for both hydrophilic and lipophilic antioxidants and gives a clearer picture of the overall capacity, not just the effect at a single point. Moreover, it has also been reported that the lower repeatability relative standard deviation of using TEAC values in different antioxidants in several different laboratories compared to those of IC50, further suggests that the use of TEAC is more effective for reducing the variance among laboratories [30].

It should also be mentioned that the lower the TEAC (Trolox Equivalent Antioxidant Capacity) value, the higher the substance’s antioxidant power, because TEAC measures how well a compound neutralizes the DPPH radical, and a faster/stronger reaction (lower value) means more effective scavenging compared to Trolox, indicating greater antioxidant capacity.

According to the results shown in Table 5, and differently to what was observed in the ABTS assay, the juice-derived TAC fractions showed higher antioxidant capacity (lower TEAC values) compared to their TLC extracts, probably due to more bioactive amphiphilic phenolics and carotenoids having been migrated in the TAC fractions of the watery juice. The TLCs of juice showed the lowest antioxidant capacity in this assay (higher TEAC values), but were still considerable (Table 5), probably due to their high content in carotenoids, particularly lycopene, which is well documented for its strong radical-scavenging properties in the DPPH assay too [28,29,31].

The differences observed in the antioxidant capacity of the samples when assessed by the ABTS and DPPH assays further suggest that individual assays are more suitable for specific classes of antioxidant compounds. In particular, certain antioxidants, such as phenolic compounds, tend to perform better under the conditions of one assay, whereas others, such as carotenoids, exhibit greater reactivity under the conditions of alternative assays. Consequently, when samples contain multiple classes of antioxidants—namely both phenolics and carotenoids—it is advisable to evaluate their antioxidant capacity using both the ABTS and DPPH methods. For this reason, both assays were employed in the present study to provide a more comprehensive assessment of the total antioxidant capacity of watermelon juice extracts and their by-products, which are rich in both phenolics and carotenoids. The results obtained further confirm the distinct responses of these two assays when applied to such complex antioxidant systems.

Regarding the by-products, their TAC fractions showed similar antioxidant capacity with that of the TAC fractions of the juice, while the TLC fractions of the by-products showed much better antioxidant capacity from the TLC of the juice. The antioxidant capacity of the TLC fraction of the by-products was even comparable to that of their TAC fractions, suggesting that the equal distribution of the phenolics between these two fractions during the CCD of the TL from the By-Products is the dominant parameter that determined the obtained values for the TEAC values of both the TAC and the TLC fractions of the By-Products, rather than their carotenoids content. Overall, again this assay suggests that the observed high antioxidant capacity of the fractions from the By-products further supports their utilization of the watermelon by-products as valuable sustainable sources of potent antioxidant natural bioactives.

### 4.6. Antioxidant Activity Based on the FRAP Assay

Apart from the free radical scavenging assays, ABTS and DPPH, for further evaluating the antioxidant capacity of our samples from a different perspective, we also conducted another assay, the FRAP (Ferric Reducing Antioxidant Power) assay, with a completely different method principle to the ABTS and DPPH methodologies. The FRAP assay was used in this study, alongside DPPH and ABTS, because each one of them measures different aspects of antioxidant activity: DPPH and ABTS primarily assess radical scavenging (electron or hydrogen donation to a radical), while FRAP specifically measures the antioxidant’s capacity to reduce ferric iron (Fe^3+^ to Fe^2+^) via electron transfer (reducing power), providing a more complete picture, especially for hydrophilic antioxidants and compounds acting through reduction. A combination of these tests reveals different antioxidant mechanisms (like radical scavenging vs. reducing power) within a complex sample, offering a fuller understanding than a single test alone, and this is why we performed this assay too in the present study.

The results of the FRAP assay (Table 6) demonstrated that the amphiphilic (TAC) extracts of both watermelon juice and by-products exhibited higher antioxidant activity compared to their neutral (TLC) counterparts, which matches with the general concept of strong antioxidant activities in the more polar amphiphilic compounds for this assay, which include the phytochemicals of fruits and their by-products, like watermelon. This finding suggests that amphiphilic samples rich in more polar bioactives—likely rich in phenolic compounds—were more effective in reducing ferric (Fe^3+^) to ferrous (Fe^2+^) ions than carotenoids and lycopene. The enhanced reducing power of these compounds can be attributed to the presence of hydroxyl (-OH) functional groups and conjugated aromatic structures, which facilitate electron delocalization and efficient electron transfer during redox reactions [20].

Conversely, the more neutral TLC extracts, particularly those derived from the by-products, exhibited lower FRAP values. This reduced activity is consistent with their limited content of more polar amphiphilic compounds like phenolic compounds, which are primarily responsible for electron-donating capacity, as well as due to the lack of amphiphilic compounds that could form liposomes to enhance the antioxidant activity of carotenoids in this more polar assay, as observed in the presence of liposomes as delivery systems for carotenoids like lycopene [32].

Overall, unlike the ABTS assay—where carotenoids contributed substantially to antioxidant potential—both the DPPH and especially the FRAP method appeared to be more responsive to polar phenolic and amphiphilic constituents of watermelon in the present study. Consequently, the FRAP results highlight that the reducing antioxidant power of watermelon-derived extracts, and especially those from the by-products, is largely governed by their amphiphilic molecular components, similarly with previously reported results [33], which further suggest their potential valorization as valuable sustainable sources of such antioxidants in several applications of functional products [34].

### 4.7. ATR-FTIR Spectroscopy of TAC Fractions from Watermelon Juice and Its By-Products

ATR-FTIR spectroscopy was subsequently employed to explore structure–activity relationships and to confirm the presence of bioactive functional groups within the amphiphilic extracts of watermelon juice and its by-products. This technique provided qualitative insights into the molecular composition of the extracts, revealing characteristic absorption bands associated with classes of compounds such as phospholipids, glycolipids, triglycerides, and lipoproteins. By identifying these functional groups, ATR-FTIR analysis served as a complementary tool to the biochemical assays, offering molecular-level evidence of the bioactive structures responsible for the observed antioxidant and anti-inflammatory activities.

Comparison of the ATR-FTIR spectra of the amphiphilic (TAC) extracts from watermelon juice and by-products (Table 7) revealed highly similar profiles, indicating a comparable chemical composition and the presence of common classes of bioactive compounds in the TAC extracts of both the juice and the by-products.

A broad absorption band between 3200 and 3500 cm^−1^ was observed in both spectra, corresponding to O–H stretching vibrations characteristic of hydroxyl groups, confirming the presence of phenolic compounds and hydrogen-bonded structures. Strong bands detected between 2850 and 3000 cm^−1^ were assigned to the asymmetric and symmetric C–H stretching vibrations of methylene (–CH_2_–) and methyl (–CH_3_) groups, indicative of long hydrocarbon chains typical of lipid constituents.

A weaker absorption feature appearing between 1640 and 1680 cm^−1^ was attributed to C=C stretching vibrations of conjugated double bonds, suggesting the presence of polyunsaturated fatty acids (PUFAs). Additional bands between 1510 and 1650 cm^−1^ were assigned to conjugated C=C polyene structures, which are characteristic of carotenoids.

In the fingerprint region, prominent peaks between 1000 and 1300 cm^−1^ corresponded to P=O stretching vibrations of phosphate groups, confirming the presence of amphiphilic phospholipids, glycolipids, and other polar lipids within the TAC extracts. Finally, the absorption bands detected between 850 and 900 cm^−1^ were attributed to P–O–C stretching modes, representing the linkage between phosphate groups and glycerol backbones, typical of glycerophospholipids.

Collectively, these spectral features substantiate that both juice and by-product TAC extracts contain similar lipid-based bioactive components—primarily phospholipids, glycolipids, carotenoids, and phenolic compounds—which together contribute to their antioxidant, anti-inflammatory, and antithrombotic properties observed in the biological assays.

### 4.8. Evaluation of Antithrombotic and Anti-Inflammatory Activities of Samples by the Platelet Aggregometry Assay

Platelet aggregometry assessment in hPRP is one of the golden standard methodologies to evaluate the induction or inhibition of platelet aggregation by a specific compound/extract. For example, this methodology has been successfully applied not only for pharmaceuticals with potent anti-platelet, antithrombotic and anti-inflammatory properties [10], but also for natural compounds and extracts that can inhibit platelet aggregation induced by classic platelet agonists like ADP or by thrombo-inflammatory mediators like PAF [4,5,6,8,15]. Inhibition of PAF reflects not only antithrombotic activity but also anti-inflammatory potential, given PAF’s central role in inflammatory signaling cascades in platelets, but also in several other cells that express in their membranes PAF-receptor, suggesting that the studied anti-PAF potency of a bioactive compound in platelets also reflects its more general anti-inflammatory potency. Overall, assessing anti-PAF effects on platelets is crucial because PAF is a potent lipid mediator driving inflammation and thrombosis, causing platelets to aggregate and release inflammatory signals, making anti-PAF agents potential treatments for cardiovascular diseases (like atherosclerosis), asthma, sepsis, and other inflammatory conditions by blocking these harmful pro-thrombotic and inflammatory pathways.

In contrast, ADP is one of the well-established platelet agonists acting on platelets through a specific for ADP receptor, by which classic platelet aggregation and thrombotic stimulation is induced. Assessing anti-ADP effects on platelets is crucial for managing cardiovascular risk, as ADP is vital for platelet aggregation (clotting), but also a target for antiplatelet drugs like clopidogrel, allowing clinicians to personalize therapy, check drug efficacy, predict thrombosis risk, and diagnose platelet disorders, ensuring patients get the right dose to prevent clots without excessive bleeding [35]. Subsequently, assessing the anti-ADP effects of natural bioactives on platelets is also crucial because, as aforementioned, ADP is a key signal for platelet activation, central to blood clot formation (thrombosis) in cardiovascular diseases like heart attacks and strokes, so finding natural compounds that block this pathway offers a way to develop safer, effective antiplatelet drugs that could reduce bleeding risks associated with current medications while preventing dangerous clots [36].

In the present study, the obtained results shown in Figure 1 and Figure 2 express the inhibitory potential of the extracts against PAF- and ADP-induced platelet aggregation in terms of IC_50_ (half-maximal inhibitory concentration), which equals to the mass in μg of the sample that when present in the aggregometer cuvette with the 250 μL of hPRP can cause 50% inhibition of the PAF-/ADP-induced aggregation of hPRP. It is important to state that the lower the IC_50_ values for a sample against either PAF or ADP induced platelet aggregation the greater its inhibitory potency against the specific thrombo-inflammatory pathway.

From all the samples assessed the TAC extracts from both the juice and the by-products showed lower IC50 values than their TLC fractions, respectively, suggesting stronger anti-inflammatory and antiplatelet potency for the amphiphilic compounds of these two sources against both PAF and ADP (Figure 1 and Figure 2). Moreover, both the anti-PAF and anti-ADP effects of the TAC fractions were almost equal, as TACs from each source showed similar IC50 values against each platelet agonist, suggesting that the anti-platelet effects of the amphiphilic natural bioactives present in these sources is not related to any specificity between these compounds for any of the two pathways assessed in this study, rather than a general anti-platelet effect by their interaction with platelets, when incubated in the hPRP for 2 min, prior to the addition of the platelet agonist.

According to Zia et al. (2021) [30], watermelon flesh contains amphiphilic phenolic compounds such as quercetin, luteolin, and gallic acid, which are known to exert PAF-inhibitory effects when isolated from other natural sources [15]. Thus, the potent anti-PAF activity of the amphiphilic extracts (TAC) from watermelon juice can be attributed to these bioactive flavonoids and phenolic acids, acting either independently or synergistically with other lipid components like bioactive polar lipids that also exert strong anti-PAF effects [4,5,6,8].

Moreover, the presence of carotenoids in these fractions not only provide antioxidant stability to all the other bioactives (i.e., phenolics and PUFA or MUFA of the polar lipids) and thus to the whole extract itself, but can also indirectly affect PAF-related inflammatory manifestations, since carotenoids—particularly lycopene—interact with PAF metabolism, to help prevent cardiovascular damage by modulating oxidative stress and inflammation, often by inhibiting PAF synthesis pathways and enhancing the body’s own defenses [37,38]. While PAF is a pro-inflammatory mediator, lycopene’s combined action with the other watermelon derived bioactive compounds helps balance its effects, reducing tissue damage and promoting heart health, especially when co-present with vitamins like vitamin E [38].

The presence of natural coumarins in watermelon, which have also been reported to inhibit PAF-induced platelet aggregation [38,39,40], may further contribute to this effect. Although coumarins from watermelon have not been directly linked to PAF inhibition, still watermelon contains natural coumarins, particularly in its seeds, which show promising antioxidant, anticancer (against breast/lung cells), and potential anti-inflammatory effects.

Overall, the high phenolic content and the polar lipids present in TAC extracts seem to be the main bioactives with anti-PAF effects, while carotenoids like lycopene provide antioxidant stability and indirect anti-PAF effects through reducing PAF synthesis.

Amphiphilic extracts from the by-products also demonstrated potent PAF inhibition, almost equal to that observed in TAC from Juice, likely due again to the presence of polar lipids and bioactive phenolics [4,6,8,31]. The polar lipid content that usually migrates to the TAC extracts during the counter current distribution methodology applied, seems to mostly contribute to the higher anti-PAF effects of the TAC extracts in the by-products compared to that of the TLC extracts from the same source.

The neutral TLC fractions in both juice and the by-product extracts likely owe their limited yet detectable PAF inhibition to lipophilic compounds of lower polarity from the classic amphiphilic bioactives, which usually migrate to petroleum ether phase during CCD, such as several free fatty acids (mostly *cis* monounsaturated fatty acids—MUFA—like oleic acid, and omega-3 polyunsaturated fatty acids—ω-3 PUFA—like eicosapentaenoic acid—EPA), cucurbitacins, sitosterols, and at some extents tocopherols, among others, which have been reported to modulate platelet aggregation and oxidative pathways to some extent, while some of them interact directly with the PAF-pathway [41,42].

With respect to the effects of all extracts against ADP-induced aggregation, previous studies have identified several phenolic bioactives like quercetin, luteolin, and gallic acid as natural inhibitors of the ADP induced aggregation of platelets [15,43,44,45], which further supports the potent anti-ADP effect observed for the TAC extracts of both the juice and the by-products. Watermelon carotenoids like lycopene and b-carotene seem to contribute directly to the anti-ADP effect of all extracts on platelets, since differently from their indirect effects on PAF (inhibition of PAF-synthesis rather than inhibition of PAF-induced platelet aggregation), these watermelon carotenoids have been reported to exhibit a concentration-dependent inhibition of platelet aggregation and the ATP-release reaction stimulated by ADP, or other platelet agonists (i.e., collagen or arachidonic acid) in both washed human platelets and PRP [46]. The PLs of the amphiphilic extracts of the by-products, enriched in ω-3 PUFAs, such as α-linolenic acid, also demonstrated measurable anti-ADP effects, consistent with literature linking their ω-3 fatty acid content to platelet inhibition and vascular protection through this pathway and through antagonizing the arachidonic acid too [47].

Overall, the TAC extracts exhibited the strongest inhibitory activity against both PAF and ADP, consistent with their composition rich in amphiphilic bioactives, including several phenolics and carotenoids, but also due to their polar lipids with polyunsaturated fatty acids as parts of their structures. Despite having moderate phenolic content, their bioactivity appears to stem primarily from bioactive phospholipids, which act synergistically with the co-present amphiphilic antioxidants (phenolics and carotenoids). Finally, the anti-PAF activities of all TAC extracts were of similar potency (IC50 values) with their anti-ADP effects, which further suggest a general and non-specific potency of these extracts to inhibit platelet aggregation, independently from the signaling pathway. These findings highlight not only the health benefits of TAC from watermelon juice, but mainly the potential of valorizing watermelon by-product TAC extracts as sources of natural antithrombotic and anti-inflammatory amphiphilic compounds suitable for being incorporated into functional foods and nutraceuticals.

### 4.9. Fatty Acid Composition of the Bioactive PLs of the TAC Extracts from Watermelon Juice and By-Products

LC–MS analysis was performed exclusively on the amphiphilic (TAC) extracts of watermelon juice (WMJ) and by-products (WMBP), as these fractions demonstrated the most potent biological activities against PAF and ADP in the platelet aggregometry assays. Combined with the results of the Folin–Ciocalteu assay, this analysis supports the conclusion that the bioactivity of the extracts primarily arises from polar lipids, which contain bound fatty acids of varying saturation/unsaturation levels.

Saturated fatty acids constituted approximately half of the total fatty acid content in both TAC extracts, accounting for 49.94 ± 0.58% in WMJ and 51.35 ± 0.55% in WMBP (Table 8). The major SFAs were palmitic (C16:0) and stearic (C18:0) acids, together representing over 95% of the SFA fraction. Palmitic acid was slightly more abundant in the by-products extract (30.34%) than in the juice (28.40%), while stearic acid displayed the opposite trend (20.78% in juice vs. 19.21% in by-products). Minor SFAs such as myristic (C14:0) and pentadecylic (C15:0) acids were detected in trace amounts (<0.5%) in both samples, whereas pelargonic (C9:0) and margaric (C17:0) acids were observed only in the by-products (0.1–0.9%). The slightly higher SFA content in the by-product extract likely reflects the greater proportion of membrane-bound structural lipids retained in the residual plant matrix following juice extraction.

The monounsaturated fraction was dominated by oleic acid (C18:1 c9, n-9), accounting for 31.28 ± 0.56% in WMJ and 15.80 ± 0.22% in WMBP. A smaller amount of palmitoleic acid (C16:1 c9, n-7) was also present, slightly higher in the by-products (1.19%) than in the juice (0.88%). Overall, total MUFA content decreased from 32.16% in juice to 16.99% in by-products. MUFAs are well recognized for their anti-platelet and anti-inflammatory effects [41,42], and thus for their cardioprotective properties and metabolic health benefits, including improved insulin sensitivity and reduced oxidative stress [48,49], while when part of the structure of polar lipids they can show dual effects; direct anti-PAF effects when bound to the polar lipids [4,5,6,8], and some more general anti-platelet and anti-inflammatory properties through other mechanisms of action (i.e., when membrane bound phospholipase A2 (PLA2) releases them from the membrane PL towards the cytoplasm where they can act as free fatty acids with the aforementioned benefits) [41,42,48,49]. Furthermore, owing to their single double bond, MUFAs exhibit greater oxidative stability than polyunsaturated fatty acids (PUFAs), thereby contributing to enhanced shelf life and stability in functional food and pharmaceutical formulations [50].

A significant increase in total PUFA content was observed in the by-product extract (31.66 ± 0.39%) compared to the juice (17.91 ± 0.17%). The n-6 PUFA linoleic acid (C18:2 c9,12) was present in both extracts and moderately higher in WMBP (16.02%) than in WMJ (13.63%). Notably, the ω-3 PUFA fraction increased sharply from 4.28 ± 0.09% in juice to 15.64 ± 0.30% in the by-products, mainly due to the higher content in the ω-3 PUFA, α-linolenic acid (ALA, C18:3 c9,12,15), which reached 14.14% in WMBP. Thus, the PLs of the by-product TAC extracts exhibited a significantly higher content of polyunsaturated fatty acids (PUFAs)—particularly ω-3 fatty acids—which have been extensively associated with anti-PAF and anti-ADP bioactivities in other natural sources too [4,5,6,8].

More specifically, ALA is an essential ω-3 PUFA known for its anti-inflammatory and cardioprotective properties [51,52,53,54,55], while when bound to polar lipids, it can also exhibit dual activities, meaning a direct anti-platelet effect of the polar lipids bearing ALA on PAF-/ADP-receptors and their signaling [4,5,6,8], as well as a more general anti-platelet and anti-inflammatory properties through other mechanisms of action involving the enzyme activity of membrane bound PLA2 and its release in the cytoplasm where it can act as a free omega-3 PUFA providing thus all the well-established benefits related to these PUFA [51,52,53,54,55].

Another two long-chain ω-3 fatty acids—eicosapentaenoic acid (EPA, C20:5 n-3) and docosahexaenoic acid (DHA, C22:6 n-3)—were also detected exclusively in the by-product extract at low but considerable amounts (0.68% and 0.82%, respectively). Despite their low concentrations, their presence contributes meaningfully to the nutritional and biofunctional value of the WMBP fraction. Similarly, to what has been observed for oleic acid, these omega-3 fatty acids, particularly ALA, EPA, and DHA, have been linked to reduced platelet aggregation and inhibition of inflammatory mediators such as PAF and ADP, especially when esterified to PLs [41,42,47,53,54,55,56,57,58,59]. The presence of these PUFAs in the PLs of the amphiphilic TAC fractions of both the juice and the by-products provides a structural basis for their dual anti-inflammatory and antithrombotic effects [59].

The ω-6/ω-3 PUFA ratio in the bioactive polar lipids of the juice was approximately 3:1, substantially lower than the typical 15:1 ratio observed in Western diets. Although ω-6 fatty acids are essential, an excess of ω-6 relative to ω-3 is associated with pro-inflammatory effects and an increased risk of chronic diseases [58,59]. Thus, the low ω-6/ω-3 ratio found in the polar lipid fractions, particularly in the WMBP extract, reflects a favorable fatty acid balance associated with anti-inflammatory and cardioprotective outcomes.

The polar lipids of watermelon by-products, in particular, exhibited the most balanced low ω-6/ω-3 PUFA ratio and the highest ω-3 PUFA content, underscoring their superior nutritional and bioactive potential. The coexistence of polar lipids rich in MUFA and ω-3 PUFA with carotenoids and phenolic compounds within these TAC extracts likely enhances both antioxidant and anti-inflammatory capacity. These findings not only rationalize the strong anti-PAF and anti-ADP activities observed experimentally but also support the valorization of watermelon by-products as a sustainable source of health-promoting amphiphilic bioactives (phenolics, carotenoids and polar lipids) that usually act synergistically and thus are suitable for use in functional foods, nutraceuticals, cosmeceuticals, and pharmaceutical formulations.

### 4.10. Structural Elucidation and Proposed Structures of the Main Bioactive Phospholipids Detected in the TAC Extracts of Both the Watermelon Juice and By-Products

LC–MS based lipidomics analysis further supports the biological findings observed for the amphiphilic TAC fractions from watermelon juice and its by-products, according to the structures of PL identified to be present in these amphiphilic mixtures. Each TAC fraction exhibited distinct lipidomic profile based on the LC-MS analysis of the non-saponified polar lipids, which are associated with their functional activities, according to structure activity relationships. More specifically, specific phospholipid subclasses, predominantly phosphatidylethanolamines (PE) and phosphatidylcholines (PC), were identified in the TAC fractions from both the juice and its by-products, with, however, the nature of the acyl chains differing significantly (Table 9), consistent with their fatty acid profile that was observed after saponification (Table 8)

PLs from the watermelon juice TAC fractions contained high levels of PE and PC species bearing MUFA (i.e., oleic acid, C18:1) or ω-PUFA, with a moderately low ω-6/ω-3 ratio (~3) (Table 9). In contrast, PLs from the watermelon by-products TAC fractions were more abundant in PEs and PCs bearing mainly esterified ω-3 polyunsaturated fatty acids (C18:3, EPA, DHA) into their structures, including ether-linked species (e.g., PE O-31:6, PE O-44:12) (Table 9). The presence of omega-3 containing PCs and PEs in the TAC of by-products comes in accordance with their elevated ω-3 PUFA content (C18:3, 20:5, 22:6) and a more favorable ω-6/ω-3 ratio (~1), as previously determined from the saponified FA pool (Table 8).

These results further support the observed potent anti-PAF and ant-ADP activities of the TAC extracts from both the juice and the by-products, providing a structural activity relationship-based explanation for the observed anti-inflammatory and antiplatelet properties. Thus, the biofunctional properties of the polar lipids (PL) extracted from watermelon appear to be governed not only by fatty acid composition but also by molecular structural architecture and synergistic interactions with other amphiphilic bioactives, allowing them to exhibit pleiotropic actions by (i) targeting thrombo-inflammatory pathways mediated by PAF and ADP, (ii) providing endogenous antioxidant protection to the oxidation-prone PUFA and MUFA moieties within the lipid matrix, (iii) by the MUFA and PUFA mediated inhibition of COX and thus by reducing pro-inflammatory eicosanoids production when these MUFA and PUFA are released from the cell membrane polar lipids by PLA2, as well as by the production of resolvins from these ω-3 PUFA, and (iv) by inducing the expression of anti-inflammatory and antithrombotic genes, while downregulating the expression of pro-inflammatory mediators like several cytokines and their downstream thrombo-inflammatory stimuli [59].

Overall, the potent anti-PAF and anti-ADP activities observed in the TAC fractions from the Watermelon Juices can be associated with the high content of monounsaturated and alpha linolenic-rich PE and PC species, in synergy with their phenolic constituents, which can act synergistically to stabilize platelet membranes and interfere with receptor-mediated signaling [60,61]. PLs containing ω3-PUFA have also shown strong antiplatelet properties, while the low ω-6/ω-3 ratio (≈3) observed in the PLs of the watermelon juice TAC fractions may further support balanced lipid–protein interactions that modulate platelet responsiveness [62].

The significant anti-PAF and anti-ADP effects by the Watermelon By-product TAC fractions can be explained by its polar lipid profile suggesting a pronounced anti-inflammatory potential, as ω-3-enriched PE and PC species are known to act as PAF receptor antagonists, while they can alter platelet membrane microdomains, and their ω-3 PUFA, when released intracellularly, serve as precursors of pro-resolving lipid mediators like resolvins [59]. More specifically, by competing with PAF for receptor binding, these lipids disrupt PAF/PAF-R–dependent thrombo-inflammatory signaling, preventing platelet activation and aggregation, which contributes to the observed antithrombotic and anti-inflammatory effects [59].

The bioactivity of phospholipids in the TAC fractions can also be attributed, in part, to the regioselective distribution of fatty acids within the glycerol backbone, particularly at the *sn*-2 position, which critically influences membrane dynamics, enzymatic interactions, and the generation of bioactive lipid mediators. Unsaturated fatty acids at the *sn*-2 position significantly influence membrane fluidity and accessibility to phospholipase A_2_ (PLA_2_), which selectively cleaves at this position to release bioactive lipid mediators such as eicosanoids. This structural configuration may enhance anti-PAF and anti-inflammatory activity by the competitive inhibition of PAF receptor binding and by modulating the generation of signaling molecules intracellularly, which can act as precursors of resolvins and/or as inhibitors of COX, as aforementioned [59].

The important pleiotropic functions of omega-3 PUFA released from phospholipids are also supported by in vivo findings [63]. For example, higher levels of platelet phospholipids n-3 PUFA were related to reduced mortality for CVD [64], while n-3 PUFA supplements are helpful in reducing platelet aggregation with reduction in ADP-induced platelet aggregation in patients with cardiovascular diseases even if this effect was not proven in healthy patients [65].

It is worth mentioning that a portion of these dietary polar lipids, following digestion, can be incorporated into lipoproteins and cellular membranes across various cell types [59,66]. This incorporation allows them to modulate inflammatory responses, enhance HDL levels and functionality, and influence cellular lipid signaling pathways associated with PAF activity, providing protective effects against thrombo-inflammatory processes implicated in cardiovascular and other chronic diseases [59,63,66]. These systemic mechanisms complement the in vitro antioxidant and antiplatelet activities observed for the TAC fractions, suggesting that dietary consumption of such lipid bioactives may confer broader cardioprotective and anti-inflammatory benefits.

On the other hand, it is also worth mentioning that in these watermelon-derived TAC extracts no bioactive glycolipids were detected, likely because their high polarity caused them to partition into the hydroalcoholic phase during the initial Bligh–Dyer extraction; it seems that it preferentially partitions highly polar lipids such as glycolipids into the water/methanol layer. Consequently, the analyzed TAC fractions predominantly reflect PE and PC species, which account for the observed antithrombotic, and anti-inflammatory activities. This result seems to provide another structure activity relationship outcome, which seems to explain why the TAC extracts of the watermelon showed lower anti-platelet and anti-inflammatory effects against ADP and PAF, respectively, compared to other similar TAC fractions from other fruits and their by-products, in which the presence of more bioactive glycolipids provided much lower IC50 values mainly against PAF, but also against ADP and thus more potent anti-inflammatory effects [4,5,6,67]

## 5. Limitations

Although this study provides novel insights into the antioxidant, anti-inflammatory, and antiplatelet activities of amphiphilic and lipophilic extracts from watermelon juice and its by-products, several limitations should be acknowledged.

First, the phytochemical characterization was restricted to total phenolic and carotenoid contents, and individual compounds were not identified or quantified due to the unavailability of appropriate calibration standards and validated chromatographic methods.

Second, while LC–MS analysis enabled the determination of fatty acid profiles and the proposal of representative phospholipid structures, comprehensive chromatographic documentation—including annotated HPLC chromatograms, UV–Vis data, and full mass spectral metadata—was beyond the scope of this work.

Third, the platelet aggregation assays relied on PAF- and ADP-induced maximal aggregation as biological reference controls, in vitro, and thus lacked in vivo assessment in a dietary intervention study for example.

Finally, the extraction and fractionation procedures, while appropriate for isolating amphiphilic and lipophilic lipid classes, are not automated to reduce errors, as can be shown by state-of-the-art HPLC methodology, and thus the full lipidome of watermelon by-products may be underrepresented (i.e., no glycolipids were detected in these extracts, when specific highly bioactive glycolipids have been detected in similar extracts from other fruits, which seem to be the main factor for the lower anti-inflammatory and antiplatelet potency detected in the watermelon derived extracts than those for the extracts from other fruits like apple and avocado).

Moreover, important general quality and safety issues that also need to be addressed prior to any potential valorization of watermelon-derived bioactives are also related to variation in the composition of watermelon and its by-products, as well as to perishability (degradation during storage/processing), processing challenges (heat sensitivity, extraction efficiency), sensory issues (particle size, taste in food products), and potential interactions (with medications like anticoagulants) or toxicological evaluation and health conditions (hypotension, kidney issues), plus general lack of awareness about safety and valorization of byproducts [68,69].

These limitations should be considered when interpreting the findings and planning future studies and especially clinical trials based on in vivo dietary intervention studies with watermelon or its extracts.

## 6. Conclusions

This study shows that watermelon juice and by-product extracts contain amphiphilic bioactives (i.e., polar lipids with MUFA or ω-3 PUFA in their structures, along with phenolic and carotenoid constituents) that contribute to measurable antioxidant and antithrombotic activities. Among the tested fractions, the amphiphilic TAC extracts demonstrated the strongest effects, exhibiting the lowest IC_50_ values against both PAF- and ADP-induced platelet aggregation. ATR-FTIR and LC–MS analyses confirmed the presence of PUFA-rich phospholipids, carotenoids, and phenolic compounds, with the by-product TAC fraction displaying a notably higher ω-3 PUFA content and a more favorable ω-6/ω-3 ratio. These compositional differences align with its enhanced biological activity. Overall, the findings highlight watermelon by-products as a valuable and sustainable source of amphiphilic bioactives with potential valorization in nutraceutical and cosmetics relevant applications.

## Figures and Tables

**Table 1:** Extraction yield for TL, TLC and TAC expressed as % (g of dry weight (DW) of extract/100 g of sample). Results are expressed as median, max and min value (*n* = 3) for all watermelon samples assessed.

Samples	Yield for TAC			TLC			TL		
	Median	Max	Min	Median	Max	Min	Median	Max	Min
Watermelon juice	0.24	0.38	0.05	0.06	0.06	0.03	0.27	0.44	0.11
Watermelon by-products	0.15	0.15	0.04	0.10	0.20	0.04	0.24	0.25	0.19

Abbreviations: TAC = extracts of total amphiphilic compounds, TLC = extracts of total lipophilic compounds, TL = extracts of total lipids.

**Table 2:** Comparison of total phenolic content (TPC) in watermelon juice and by-products, expressed as mg Gallic Acid Equivalent (GAE)/g extract, for both total amphiphilic compounds (TAC) and total lipophilic compounds (TLC) of all watermelon samples assessed. Results are expressed as median, max and min value (*n* = 3).

Samples	TPC of TACs *			TPC of TLCs *		
	Median	Min	Max	Median	Min	Max
Watermelon juice	40.73	13.62	60.85	85.23	60.58	112.30
Watermelon by-products	25.92	24.34	51.62	20.65	7.74	92.57

* mg GAE/g DW of extract (DW = Dry Weight).

**Table 3:** Comparison of total carotenoid content (TCC), expressed as mg Carotenoid Equivalent (CE)/g extract, for both total amphiphilic compound (TAC) and total lipophilic compound (TLC) extracts of all watermelon samples assessed. Results are expressed as median, max and min value (*n* = 3).

Samples	TCC of TACs ^1^			TCC of TLCs ^1^		
	Median	Min	Max	Median	Min	Max
Watermelon juice	2.88	2.80	7.78	17.30 *	13.61	21.84
Watermelon by-products	3.14	2.83	13.62	2.49	1.40	6.84

^1^ mg CE/g DW of extract (DW = Dry Weight); * denotes statistically significant difference in the TCC of the TLC fractions of the juice when compared to the TCC of all the other samples assessed (*p* < 0.05 in all these comparisons according to the Kruskal–Wallis test).

**Table 4:** Comparison of antioxidant capacity, expressed as ABTS values (μmol Trolox Equivalent (TE)/g dry weight (DW) of extract), for total amphiphilic compounds (TAC) and total lipophilic compounds (TLC) in watermelon juice and its by-products.

Samples	ABTS Values of TACs ^1^			ABTS Values of TLCs ^1^		
	Median	Min	Max	Median	Min	Max
Watermelon juice	3.70	3.60	5.60	7.79 *	7.76	14.11
Watermelon by-products	1.33	0.59	1.76	3.85 *	1.66	8.72

^1^ μmol TE/g DW of extract (DW = Dry Weight); * denotes statistically significant difference in the ABTS value of the TLC fractions compared to the TAC fractions, both in the juice and the By-Products (*p* < 0.05 in these comparisons according to the Kruskal–Wallis test).

**Table 5:** Comparison of trolox equivalent antioxidant capacity (TEAC), as measured by the DPPH assay (expressed as TEAC values), for total amphiphilic compounds (TAC) and total lipophilic compounds (TLC) in watermelon juice and watermelon by-products.

Samples	DPPH-TEAC Values of TACs ^1^			DPPH-TEAC Values of TLCs ^1^		
	Median	Min	Max	Median	Min	Max
Watermelon juice	0.0049	0.0030	0.0099	0.0190 *	0.0160	0.0380
Watermelon by-products	0.0034	0.0025	0.0100	0.0046	0.0025	0.0130

^1^ TEAC values = IC50 of Trolox (μg/L)/IC50 of the sample (μg/L); * denotes statistically significant difference in the lower antioxidant capacity (higher TEAC values) of the TLC fraction from the juice compared to all the other samples assessed (*p* < 0.05 in all these comparisons according to the Kruskal–Wallis test).

**Table 6:** Comparison of antioxidant capacity, as measured by the FRAP assay (μmol TE/g Dry Weight (DW) of extract), for total amphiphilic compounds (TAC) and total lipophilic compounds (TLC) in watermelon by-products.

Samples	FRAP Values of TACs ^1^			FRAP Values of TLCs ^1^		
	Median	Min	Max	Median	Min	Max
Watermelon juice	2.11	1.11	4.32	1.38	0.73	1.82
Watermelon by-products	1.66	1.48	4.90	0.20 *	0.14	0.27

^1^ μmol TE/g DW extract (DW = Dry Weight); * denotes statistically significant difference in the lower antioxidant capacity (higher TEAC values) of the TLC fraction from the by-products compared to all the other samples assessed (*p* < 0.05 in all these comparisons according to the Kruskal–Wallis test).

**Table 7:** Characteristic infrared (IR) absorption peaks for the TAC extracts of watermelon by-products and watermelon juice, with corresponding functional group assignments.

Peak (cm^−1^)	TACs of Watermelon Juice	TACs of Watermelon By-Products	Bond/Functional Group Correlation
3200–3500	+	+	O–H (hydroxyl) bonds, characteristic of this functional group in phenolic compounds, Glycolipids bearing a hydroxyl head group and fatty acids
2850–3000	+	+	C–H (alkyl), typically from fatty acids and aliphatic compounds like those present in Polar Lipids (PL)
1640–1680	+	+	Conjugated C=C bonds of polyunsaturated fatty acids (PUFA)
1540–1650	+	+	Conjugated polyene chain, characteristic of carotenoids|C=C stretching
1000–1300	+	+	P=O stretching characteristic of phosphoglycerolipids
850–900	+	+	P–O–C characteristic of the bond between the phosphate group and glycerol in phosphoglycerolipids

+ indicates that these peaks were indeed detected.

**Table 8:** Fatty acid profile of the PL present in TAC extracts from both watermelon juice and by-products after saponification, expressed for each fatty acid (FA) as percentage composition of total fatty acids in each sample evaluated (mean ± standard deviation (SD), *n* = 3).

Empirical Name	Fatty Acids	Watermelon Juice (%)	Watermelon By-Products (%)
Pelargonic	C9:0	ND	0.10 ± 0.02
Myristic	C14:0	0.36 ± 0.04	0.40 ± 0.04
Pentadecylic	C15:0	0.39 ± 0.03	0.38 ± 0.12
Palmitic	C16:0	28.40 ± 0.33	30.34 ± 0.41
Palmitoleic	C16:1 c9 (n7 MUFA)	0.88 ± 0.16	1.19 ± 0.107
Margaric	C17:0	ND	0.89 ± 0.13
Stearic	C18:0	20.78 ± 0.36	19.21 ±0.15
Oleic	C18:1 c9 (n9 MUFA)	31.28 ± 0.56	15.80 ± 0.22
Linoleic	C18:2 c9,12 (n6 PUFA)	13.63 ± 0.11	16.02 ± 0.15
α Linolenic	C18:3 c9,12,15 (n3 PUFA)	4.28 ± 0.09	14.14 ± 0.26
EPA	C20:5 c5,8,11,14,17 (n3 PUFA)	ND	0.68 ± 0.01
DHA	C22:6 c4,7,10,13,16,19 (n3 PUFA)	ND	0.82 ± 0.04
SFA		49.94 ± 0.58	51.35 ± 0.55 *
UFA		50.10 ± 0.58	48.66 ± 0.55
MUFA		32.16 ± 0.42 **	16.99 ± 0.29 **
PUFA		17.91 ± 0.17	31.66 ± 0.39
n3PUFA		4.28 ± 0.09	15.64 ± 0.30
n6PUFA		13.63 ± 0.11	16.02 ± 0.15
n6/n3		3.19 ± 0.07	1.02 ± 0.02
UFA/SFA		1.00 ± 0.02	0.95 ± 0.02

Abbreviations: EPA = Eicosapentaenoic acid; DHA Docosahexaenoic acid; n3 = omega-3; n6 = omega-6; n9 = omega-9; SFA = saturated fatty acids; MUFA = monounsaturated fatty acids; PUFA = polyunsaturated fatty acids; ND = undetectable (usually defined as fatty acids detected with a contribution of less than 0.005% to the total fatty acid content). * indicates statistically significant difference (*p* < 0.05) of SFA compared to UFA. ** indicates statistically significant difference (*p* < 0.05) of MUFA compared to PUFA.

**Table 9:** Proposed structures of the PLs detected in the TAC fractions of both Watermelon Juice and By-Products.

Main Class of PL	Elution Time (min)	*m*/*z*	Representative Molecular Species	Proposed Structures
PE	7.6	634.8736	PE O-29:0	PE O-15:0|14:0
	9.2	634.8928	PE O-29:0	PE O-15:0|14:0
	9.2	714.844	PE 34:2	PE 16:0|18:2 or PE 16:1|18:1
	9.2	714.8397	PE 34:2	PE 16:0|18:2 or PE 16:1|18:1
	10	634.8897	PE O-29:0	PE O-15:0|14:0
	10.27	634.8903	PE O-29:0	PE O-15:0|14:0
	10.27	698.4154	PE 33:3	PE 15:0|18:3
PC	9.2	770.8635	PC 36:2	PC 18:1|18:1
	9.6	714.8397	PC O-33:2	PC O-15:0|18:2
	9.6	770.8613	PC 36:2	PC 18:0|18:2
	10.27	714.4099	PC 32:2	PC-14:0|18:2
	10.27	770.8603	PC 36:2	PC-18:0|18:2
**Main** **class of PL**	**Elution Time (min)**	***m*** **/** ***z***	**Representative Molecular Species**	**Proposed structures**
PE	7.17	734.0131	PE 36:6	PE 18:3|18:3
	7.55	734.015	PE 36:6	PE 18:3|18:3
	7.55	820.1884	PE O-44:12	PE O-22:6|22:6
	8.38	734.0216	PE 36:6	PE 18:3|18:3
	8.56	734.0174	PE 36:6	PE 18:3|18:3
	8.74	734.0134	PE 36:6	PE 18:3|18:3
	8.74	744.9631	PE 36:1	PE 18:0|18:1
	9.159	666.0286	PE O-31:6;O	PE O-9:0|22:6
	9.159	734.0236	PE 36:6	PE 18:3|18:3
	9.656	714.5567	PE O-35:2	PE O-17:0|18:2
	10.245	734.3987	PE 36:6	PE 18:3|18:3
PC	7.17	692.1602	PC 31:6	PC 9:0|22:6
	7.55	666.028	PC 29:5	PC 9:0|20:5
	7.55	820.1884	PC 39:6;O	PC 17:0|22:6
	7.55	744.9631	PC 34:1	PC 16:0|18:1
	7.55	750.5295	PC O-36:5	PC O-16:0|20:5
	10.651	666.026	PC 29:5	PC 9:0|20:5
	10.817	666.0278	PC 29:5	PC 9:0|20:5
	12.177	666.028	PC 29:5	PC 9:0|20:5

## Data Availability

The original contributions presented in this study are included in the article. Further inquiries can be directed to the corresponding author.

## Abbreviations

**Abbreviation**	**Meaning**
**EPA**	Eicosapentaenoic acid
**DHA**	Docosahexaenoic acid
**n3**	Omega-3
**n6**	Omega-6
**n9**	Omega-9
**SFA**	Saturated fatty acid
**MUFA**	Monounsaturated fatty acid
**PUFA**	Polyunsaturated fatty acid
**ND**	Undetectable
**TAC**	Total amphiphilic compound
**TLC**	Total lipophilic compound
**TL**	Total lipid
**WMJ**	Watermelon juice
**FFA**	Free fatty acid
**LC–MS**	Liquid chromatography–mass spectrometry
**KOH**	Potassium hydroxide
**HPLC**	High-performance liquid chromatography
**ESI**	Electrospray ionization
**m/z**	Mass-to-charge ratio
**TPC**	Total phenolic content
**TCC**	Total carotenoid content
**TAA**	Total antioxidant activity
**DPPH**	2,2-diphenyl-1-picrylhydrazyl
**ABTS**	2,2′-azino-bis(3-ethylbenzothiazoline-6-sulfonic acid)
**FRAP**	Ferric reducing antioxidant power
**TE / TEAC**	Trolox equivalent / Trolox equivalent antioxidant capacity
**DW**	Dry weight
**PRP**	Platelet-rich plasma
**PPP**	Platelet-poor plasma
**PAF**	Platelet-activating factor
**ADP**	Adenosine diphosphate
**IC_50_**	Half-maximal inhibitory concentration
**ATR–FTIR**	Attenuated total reflectance Fourier-transform infrared spectroscopy
**CCD**	Counter-current distribution
**GAE**	Gallic acid equivalent
**CE**	β-Carotene equivalent
**HCl**	Hydrochloric acid
**Na_2_CO_3_**	Sodium carbonate
**PLA_2_**	Phospholipase A_2_
**HDL**	High-density lipoprotein
***sn*-2**	Stereospecific numbering position 2 on glycerol
**PL**	Polar lipid
**FA**	Fatty acid
**UFA**	Unsaturated fatty acid
**hPRP**	Human platelet-rich plasma
